# A comparison of feature selection and classification methods in DNA methylation studies using the Illumina Infinium platform

**DOI:** 10.1186/1471-2105-13-59

**Published:** 2012-04-24

**Authors:** Joanna Zhuang, Martin Widschwendter, Andrew E Teschendorff

**Affiliations:** 1 Statistical Genomics Group, Paul O'Gorman Building, UCL Cancer Institute, University College London, 72 Huntley Street, London WC1E 6BT, UK; 2 Department of Women's Cancer, UCL Elizabeth Garrett Anderson Institute for Women's Health, University College London, Room 340, 74 Huntley Street, London WC1E 6 AU, UK

**Keywords:** DNA methylation, Classification, Feature selection, Beadarrays

## Abstract

**Background:**

The 27k Illumina Infinium Methylation Beadchip is a popular high-throughput technology that allows the methylation state of over 27,000 CpGs to be assayed. While feature selection and classification methods have been comprehensively explored in the context of gene expression data, relatively little is known as to how best to perform feature selection or classification in the context of Illumina Infinium methylation data. Given the rising importance of epigenomics in cancer and other complex genetic diseases, and in view of the upcoming epigenome wide association studies, it is critical to identify the statistical methods that offer improved inference in this novel context.

**Results:**

Using a total of 7 large Illumina Infinium 27k Methylation data sets, encompassing over 1,000 samples from a wide range of tissues, we here provide an evaluation of popular feature selection, dimensional reduction and classification methods on DNA methylation data. Specifically, we evaluate the effects of variance filtering, supervised principal components (SPCA) and the choice of DNA methylation quantification measure on downstream statistical inference. We show that for relatively large sample sizes feature selection using test statistics is similar for M and β-values, but that in the limit of small sample sizes, M-values allow more reliable identification of true positives. We also show that the effect of variance filtering on feature selection is study-specific and dependent on the phenotype of interest and tissue type profiled. Specifically, we find that variance filtering improves the detection of true positives in studies with large effect sizes, but that it may lead to worse performance in studies with smaller yet significant effect sizes. In contrast, supervised principal components improves the statistical power, especially in studies with small effect sizes. We also demonstrate that classification using the Elastic Net and Support Vector Machine (SVM) clearly outperforms competing methods like LASSO and SPCA. Finally, in unsupervised modelling of cancer diagnosis, we find that non-negative matrix factorisation (NMF) clearly outperforms principal components analysis.

**Conclusions:**

Our results highlight the importance of tailoring the feature selection and classification methodology to the sample size and biological context of the DNA methylation study. The Elastic Net emerges as a powerful classification algorithm for large-scale DNA methylation studies, while NMF does well in the unsupervised context. The insights presented here will be useful to any study embarking on large-scale DNA methylation profiling using Illumina Infinium beadarrays.

## Background

DNA methylation (DNAm) is one of the most important epigenetic mechanisms regulating gene expression, and aberrant DNAm has been implicated in the initiation and progression of human cancers [1,2]. DNAm changes have also been observed in normal tissue as a function of age [3-8], and age-associated DNAm markers have been proposed as early detection or cancer risk markers [3,6-8]. Proper statistical analysis of genome-wide DNA methylation profiles is therefore critically important for the discovery of novel DNAm based biomarkers. However, the nature of DNA methylation data presents novel statistical challenges and it is therefore unclear if popular statistical methods used in the gene expression community can be translated to the DNAm context [9].

The Illumina Infinium HumanMethylation27 BeadChip assay is a relatively recent high-throughput technology [10] that allows over 27,000 CpGs to be assayed. While a growing number of Infinium 27k data sets have been deposited in the public domain [3,4,11-15], relatively few studies have compared statistical analysis methods for this platform. In fact, most statistical reports on Infinium 27k DNAm data have focused on unsupervised clustering and normalisation methods [16-19], but as yet no study has performed a comprehensive comparison of feature selection and classification methods in this type of data. This is surprising given that feature selection and classification methods have been extensively explored in the context of gene expression data, see e.g. [20-33]. Moreover, feature selection can be of critical importance, as demonstrated by gene expression studies, where for instance use of higher order statistics has helped identify important novel cancer subtypes [24,34]. Given that the high density Illumina Infinium 450k methylation array is now starting to be used [10,35] and that this array offers the coverage and scalability for epigenome wide association studies (EWAS) [36], it has become a critical and urgent question to determine how best to perform feature selection on these beadarrays.

The Illumina Infinium assay utilizes a pair of probes for each CpG site, one probe for the methylated allele and the other for the unmethylated version [10]. The methylation level is then estimated, based on the measured intensities of this pair of probes. Two quantification methods have been proposed to estimate the methylation level: (i) β-values and (ii) M-values. While the β-value measures the percentage of methylation at the given CpG site and is the manufacturer's recommended measure to use [10], the M-value has recently been proposed as an alternative measure [19]. The M-value is defined by the log2 ratio of the intensities of the methylated to unmethylated probe and has also been used in the context of other methylation array technologies [37]. The M-value is also an analogue of the quantity which has been widely used in expression microarray analysis, although there are two important differences: in the Infinium DNAm (type I) assay, both probes are (i) always measured in the same colour channel, thus dye bias does not need to be adjusted for, and (ii) the two probes are measured in the same sample. A recent study based on a titration experiment compared β and M-values and concluded that M-values, owing to their more homoscedastic nature (i.e. variance being independent of the mean), were a better measure to use [19]. However, this study was limited to one data set and feature selection was only investigated using fold-changes, while statistics and P-values were not considered.

The purpose of our study is therefore two-fold: (i) to provide a comprehensive comparison of the performance of β and M-value measures in the context of popular feature selection tools that use actual statistics to rank features (CpGs), and (ii) to compare the performance of some of the most popular feature selection and classification methods on DNAm data from different tissues and correlating with different phenotypes, so as to gain objective insights into how best to perform feature selection and classification in this novel context. To address these goals, we make use of 7 independent sets of human DNA methylation data, all generated with the 27K Illumina Infinium platform, encompassing over 1000 samples and representing over 29 million data points.

## Materials and methods

### Data sets

The 7 human DNA methylation data sets are summarised in Table 1. All the data were generated using the Illumina Infinium HumanMethylation27 BeadChip assay that enables the direct measurement of methylation at over 27,000 individual cytosines at CpG loci located primarily in the promoter regions of 14,495 unique genes. All data sets, except the TCGA lung cancer set, followed the same quality control and normalisation strategy. Briefly, non background-corrected data was used and only CpGs with intensity detection P-values less than 0.05 (i.e. significantly higher intensity above the background determined by the negative controls) were selected. Inter-array normalisation correcting for beadchip effects and variations in bisulfite conversion efficiency were performed using a linear model framework with explicit adjustment for these factors, but only if these factors were significantly correlated with PCA/SVD components.

**Table 1:** The Illumina Infinium 27 k data sets

*Data Set*	*#CpGs*	*Size*	*Tissue*	*#(N, C)*	*Age range*	*Reference*
UKOPS	25,642	261	blood	(148,113)	50-84	GSE19711
ENDOM	25,998	87	tissue	(23,64)	32-90	GSE33422
CERVX	26,698	63	tissue	(15,48)	25-92	GSE30760
OVC	27,578	177	tissue	(0,177)	25-89	[3]
T1D	22,486	187	blood	(187,0)	24-74	GSE20067
BC	24,206	136	tissue	(23,113)	31-90	GSE32393
LC	23,067	151	tissue	(24,127)	NA	TCGA

The 7 independent 27k DNAm data sets used in this paper: UKOPS - UK Ovarian Cancer Population Study: whole blood samples from women with ovarian cancer and age-matched healthy controls; ENDOM - normal endometrium and endometrial cancer; CERVX - normal cervix and cervical cancer; OVC - ovarian cancer tissue; T1D - type 1 diabetes: whole blood samples from type-1 diabetics; BC - normal breast tissue and breast cancer; and LC - normal lung tissue and lung cancer. Number of CpG probes passing quality control, total number of samples, tissue type, number of normal/cancer samples, age-range and reference to data are given. NA: not available.

### Definition of β-value and M-value

In the Illumina Infinium Human Methylation 27k BeadChip assay, bisulphite (BS) converted DNA is amplified, fragmented and hybridised to the BeadChip arrays, with each chip accommodating 12 samples as designated by Sentrix positions A-L. Each interrogated locus is represented by specific oligomers linked to two bead types, with one representing the sequence for methylated DNA (*methy*) and the other for unmethylated DNA (*unmethy*). The methylation status can be measured either by β-values or M-values.

For each specific CpG site, the β-value is calculated from the intensity of the *methy *and *unmethy *alleles, as the ratio of the fluorescent signals:

(1) $$\beta =\text{Max}\left(methy,0\right)/\left[\text{Max}\left(methy,0\right)+\text{Max}\left(unmethy,0\right)+100\right].$$

The β-value is a continuous variable between 0 (absent methylation) and 1 (completely methylated) representing the ratio of the methylated allele to the combined locus intensity. In contrast, the DNA methylation M-value is calculated as the log2 ratio of the intensities of the methylated probe to the unmethylated probe:

(2) $$\text{M}=\text{log2}\left(\left[\text{Max}\left(methy,0\right)+1\right]/\left[\text{Max}\left(unmethy,0\right)+1\right]\right).$$

The M-value is therefore a continuous variable which can in principle take on any value on the real line.

Since most interrogated CpG sites have intensities (*methy *+ *unmethy*) larger than 1,000, the offset values (i.e. 100 in β-value and 1 in M-value) have only a small effect on both measurements. Hence, the relationship between the β and M-values is approximately logistic:

(3) $$\beta =\left(2^{\wedge }\text{M}\right)/\left(2^{\wedge }\text{M}+1\right);\text{M}=\text{log}2\left(\beta /1-\beta \right).$$

While the β-value has a more intuitive biological interpretation it suffers from severe heteroscedasticity (the intrinsic variability is much lower for features which are either unmethylated or methylated, with hemi-methylated features exhibiting maximal variance) [9]. In contrast, the M-value is not directly interpretable in terms of a methylation percentage, but is more statistically valid for analysis of differential methylation levels owing to its more homoscedastic nature [19].

### Evaluation of feature selection methods

We use a multiple training-test set strategy [31] to compare the true positive detection rates of three different feature selection methods: (i) without filtering (WF), (ii) filtering based on variability (VF), (iii) filtering using supervised principal components (SPCA). Our justification for focusing on VF and SPCA is that both methods have been very popular and effective in the context of gene expression studies [21,26,27,38]. The performance measure we use to compare the different feature selection methods is the estimated positive predictive value (PPV). That is, for each method we select features significantly associated with a phenotype of interest using a training set, and these features are then evaluated in the test-set. Those that are also significant in the test set are called true positives, so the PPV measures the probability that a feature declared to be positive in the training set is a true positive. We point out that strictly speaking a true positive can only be a feature which has been shown to be positive using a gold-standard technology. However, in the absence of such gold-standard data, the training/test set strategy advocated here provides a suitable evaluation framework, since a feature that cross-validates is more likely to represent a true positive than one that does not. Moreover, this strategy has already been successfully used in previous work, see e.g. [21,25].

Thus, for each of the data sets in this study, 50 independent training and test sets (training and test set are always of equal size to ensure that P-values are comparable) were randomly generated ensuring similar demographics for the phenotype of interest in training and test set. For each training set, a supervised analysis is used to evaluate the association between each CpG methylation profile (both β-value and M-value) and the phenotype of interest. In the case of a continuous phenotype (e.g. age) the association is carried out under a linear regression model framework, while for a binary phenotype (e.g. cancer/normal status) we use t-statistics. Test statistic, p-value, q-value [39], absolute difference in means, and variance of each CpG are recorded.

Variance-filtering (VF) is a feature selection method that filters the CpGs based on their variability before the supervised analysis is performed [27,38]. The steps are

I. In each training set, select the 5,000 most variable CpGs;

II. Rank the 5,000 most variable CpGs according to the significance of their p-values (from the supervised analysis) and retain the top ranked 1,500 CpGs (or the number with p < 0.05 if this is smaller). (We note that for some studies in the diagnostic setting we retained all 5,000 CpGs to allow for comparable PPV values between studies).

III. Using stepwise evaluation sets (starting with 50 top CpGs and incrementing in steps of 50 until all top ranked CpG sites from step II are included in the evaluation set), count the number of true positives: i.e. how many CpGs have a statistic p-value < 0.05 in the corresponding test set while exhibiting the same directional change (sign of statistic) as that in the training set;

IV. For each evaluation set, the positive predictive value (PPV) is defined as the percentage of CpGs that are true positives.

Supervised PCA (SPCA) is a powerful semi-supervised approach for feature selection and classification, first proposed in [21]. Here we adapt it to the feature selection context. The steps are:

I. In each training set, perform the supervised analysis and rank all CpGs according to the significance of their p-values;

II. Select the top ranked 5,000 CpGs;

III. Run singular value decomposition (SVD) analysis on the top ranked 5,000 CpGs and find the principal component most correlated to the phenotype of interest;

IV. Rank the 5000 CpGs according to the absolute value of their corresponding coefficient in the selected principal component and further select the 1,500 CpGs with the largest absolute weights;

V. Use the stepwise evaluation sets and count, for each evaluation set size, the number of true positives in the test set using the same criteria as for the VF method;

VI. For each evaluation set, compute the PPV as described for VF method.

In the without filtering (WF) method we perform for each training set the supervised analysis on all ~27,000 CpGs (i.e. all those that pass quality control), select the top 1500, and then perform the same stepwise evaluation set analysis to estimate PPVs from the p-values in the corresponding test set.

The result of applying each of these 3 methods is a matrix of PPV values with rows labelling the evaluation set size (50 to 1500 in increments of 50) and columns labelling the 50 different runs (i.e. the 50 different training-test set partitions).

### Choice of evaluation set size

Given that different studies may exhibit widely different operating characteristics (e.g. widely different false discovery rates), even in relation to the same phenotype of interest, we decided that a more objective comparison of different feature selection methods across studies would be facilitated by choosing evaluation set sizes in each study that result in broadly similar PPV values *across studies*. Hence, evaluation set sizes were chosen so as to minimise differences in PPV between studies, subject to reasonable constraints on the minimum and maximum set sizes.

### Comparison of classification algorithms

We use the same multiple training-test partition strategy (50 runs) as used in our feature selection comparison, to evaluate different classification algorithms. Thus, within each study, training and test sets were always of the same size and were balanced for the phenotype of interest. We focused on four different powerful classification algorithms, which have been popular in the gene expression field: (i) Supervised PCA (SPCA) [21], (ii) the LASSO algorithm [40], (iii) the Elastic Net (ELNET) [32] and (iv) Support Vector Machines (SVM) [33,41]. We used the implementations of these algorithms as provided in the R-packages *superpc, glmnet *and *e1071*. In the case of SPCA we considered classifiers built from 1 up to 3 principal components, and the number of features (minimum was set to 10 and maximum to 5000) was optimised in the training sets using internal 10-fold cross-validation. LASSO is a special case of the Elastic Net with the penalty parameter α = 1 [32]. The Elastic Net itself was run with α = 0.05 (this choice was motivated by good performance obtained on an independent 27k DNA methylation data set, unpublished data). The additional penalty ELNET parameter λ was estimated in the training sets using internal 10-fold cross-validations. The SVM was run with a radial kernel with parameter γ = 3, and with error term ε = 0.1. These SVM parameters were fixed and not optimised, since we wanted to compare the algorithms at the same level of computational complexity (i.e. similar computational times to run). We focused on age as the phenotype of interest since it represents a challenging scenario of small effect sizes and yet it is also well established that age has a significant impact on DNA methylation patterns [3-5,42]. Since age is a continuous variable, SVM was run in regression mode, and thus for all methods the predictor is a continuous score. To evaluate concordance of age with the predictor in the test sets we used the C-index (R package *Hmisc*).

### Comparison of dimensional reduction algorithms

We considered two popular dimensional reduction algorithms: singular value decomposition (SVD) [43-46], and non-negative matrix factorization (NMF) [47-51]. In this work we use the SVD implementation as computed by the LAPACK/LINPACK routine available in R http://www.r-project.org. The application of NMF to DNA methylation is justified due to the positivity of beta-valued data. To perform NMF, the "NMF" R package [52] was used. NMF was run using the "brunet" algorithm and initialised using non-negative double SVD (NNDSVD). Hence, using this NNDSVD initialisation NMF yields the same solution under repeated runs and allows for a direct comparison to SVD. The evaluation of the 2 algorithms was performed by comparing the correlations of the inferred components with the phenotypes of interest (i.e. age and cancer diagnosis), specifically we derived and compared the corresponding R^2 ^values under a linear regression model. In the case of NMF, we needed to specify the number of components to infer. This number was estimated from the SVD analysis by comparing the spectral eigenvalues to the corresponding ones obtained by randomly permuting elements in the data matrix. This number was also the number of SVD components used when evaluating correlations with the phenotype of interest.

## Results

### The signal to noise and signal strength landscape of DNA methylation studies

**Figure 1:**
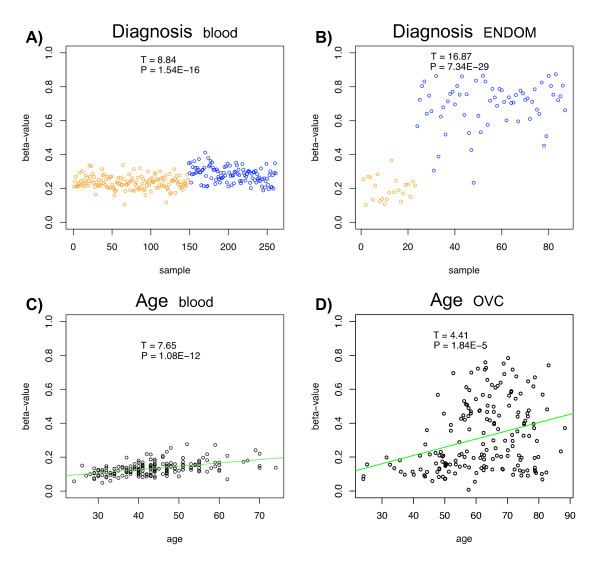
**Example beta-value methylation profiles of top ranked cancer diagnostic associated CpGs in A) UKOPS (whole blood) (cg20792833) and B) ENDOM (endometrial tissue) (cg19664945) with the x-axis labelling the sample and the cancer (blue) and normal (orange) status**. y-axis labels the beta-value. Similarly, example beta-value methylation profiles of top ranked age-associated CpGs in **C) **T1D (whole blood) (cg22736354) and **D) **OVC (ovarian cancer tissue) (cg25763788), with x-axis labeling the age of the samples. In all 4 panels we provide the *t*-test statistic and P-values of association between the beta-value and the phenotype of interest.

One of the aims of this study is to compare different feature selection and quantification methods in relation to important study-specific parameters. One of these parameters is the typical effect size of the study. In the case of a binary phenotype, the effect size of a CpG is defined, loosely speaking, as the ratio of the difference in means between the two phenotypes to a pooled standard deviation. In other words, it can be thought of as a signal to noise ratio, where the noise captures both biological and technical variation. Another important parameter to take into consideration in any association analysis is the number of features with an effect size larger than some significance threshold. Thus, in this study we ask if the effect size of CpGs associated with a phenotype of interest ("signal to noise ratio"-SNR) and their number ("signal strength") have an impact on the performance of the different feature selection methods and if this depends on the methylation measure (i.e. beta or M-values) used. In order to consider a wide range of different effect sizes and signal strengths, we considered two main phenotypes: cancer/normal status (diagnostic setting) and age; and two different tissue types: epithelial tissue and whole blood. The corresponding 27k data sets used in this study are summarised in Table 1. We verified that these studies exhibited a wide range of different effect sizes and signal strengths, depending largely on tissue type (Figure 1, Table 2). For instance, we observed, as expected, that cancer diagnostic DNAm markers in epithelial tissue have large SNRs and signal strengths, in contrast to cancer diagnostic markers in blood, which, while numerous, were characterised by much smaller effect sizes (Figure 1A-B). This fits in with the expectation that cancer associated changes in whole blood reflect mostly changes in the underlying blood cell type composition [11]. In stark contrast to diagnostic markers, we observed that age has a relatively much weaker impact on DNAm patterns (Figure 1C-D), and therefore estimated effect sizes were also smaller (Table 2). The signal strength of age-associated CpGs also varied depending on the tissue in which they were measured (Table 2).

**Table 2:** Study specific signal strengths and effect sizes

*CANCER/NORMAL*		
**Tissue**	FDR < 0.05	Effect Size
ENDOM	6967	2.57
CERVX	13616	3.45
BC	10676	1.77
LC	22239	2.19
**Blood**	FDR < 0.05	Effect Size
UKOPS	3992	1.02

***AGE***		

**Tissue**	FDR < 0.05	Effect Size
OVC	57	0.49
CERVX	0	0.69
ENDOM	0	0.68
BC	591	0.72
**Blood**	FDR < 0.05	Effect Size
UKOPS	3	0.53
T1D	294	0.61

The signal strength (measured by the number of CpGs passing an FDR threshold < 0.05) and the average effect size (Cohen's d = difference in means divided by pooled standard deviation) of the top 100 ranked CpGs for diagnosis and age-settings and for epithelial and blood tissue. Beta-values were used to estimate effect sizes. In the case of age, samples were divided into two groups by the median.

### M-values significantly outperform β values but only in the small sample size limit

A previous study based on a titration experiment advocated the use of M-values over β values, since M-values exhibit less heteroscedasticity [19]. However, this study only used fold-changes to rank features. It is therefore unclear if results differ had the features been ranked using statistics. Thus, we asked if using β-values or M-values to quantify methylation has a differential impact on the statistics of differential methylation and on the positive predictive value (PPV).

First, we verified that in our data sets, β values were highly heteroscedastic, with very low variability at the extremes of the beta-scale, while M-values were approximately homoscedastic (Additional file 1). Using t-statistics and q-values to estimate the false discovery rate (FDR) at different p-value thresholds and using all available samples (see Table 1), we observed however that the difference in FDRs between β and M-values within each study was very minor (Additional file 2). Next, we compared the PPVs obtained using either β or M-values using a multiple 50% training 50% test set strategy where features were ranked according to their t-statistics (Materials & Methods). Because for a given methylation measure we observed substantial differences in FDR rates *between studies*, in some cases even between studies that looked at the same phenotype of interest and tissue type, we decided to choose evaluation set sizes specific to each study to ensure that PPV values were as similar across studies as possible (Materials & Methods, Additional file 3).

**Figure 2:**
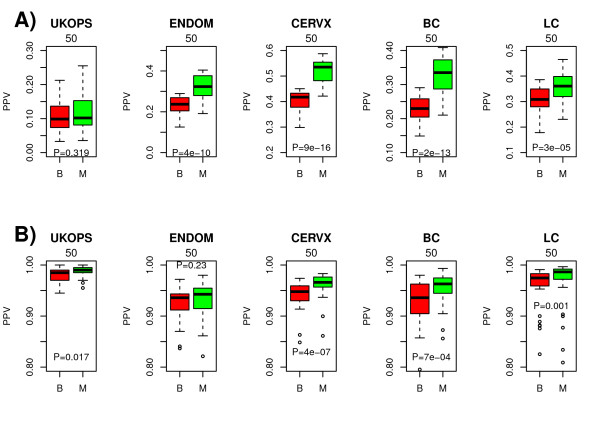
**A) PPV comparison between M-values and β-values in the small sample size limit and in the diagnostic setting**. Evaluation set sizes were the top 200, 1500, 2500, 1500, and 3000 diagnosis-associated-CpGs for UKOPS, ENDOM, CERVX, BC and LC, respectively, ranked according to regularized t-statistics. Sample sizes were 3 per phenotype (UKOPS), and 2 per phenotype (all others). **B) **As A) but now in the large sample size limit where 50% training/test partitions were used to estimate the PPV. In both A) and B), the boxplots represent the distribution of the PPV over 50 random training/test set partitions. Wilcoxon rank sum test P-values are given.

In most studies there was no appreciable difference in the PPV between β and M- values and the absolute magnitude of the *t*-test statistics of the top ranked CpGs were also very similar (Additional file 4). To study the dependency on sample size we considered again a multiple training-test set strategy but this time using training and test sets where the number of samples of each phenotype was low (either 2 to 3 samples). Since ordinary t-statistics can not be used in this small sample size limit, we used instead a popular empirical Bayes approach to rank features according to a regularized t-statistic [53]. In this small sample size limit, we observed that the PPV was significantly improved when using M-values, in some instances by at least 10% (Figure 2A). Interestingly, using 50% training/test partitions, corresponding to the largest possible sample sizes, the PPV for M-values was still higher than for β-values, but only by at most 2% (Figure 2B). However, for larger sample sizes, PPV values were close to 1 and thus differences between M-values and β-values would naturally be smaller. Thus, the improved performance of M-values over β-values is mainly due to the use of regularized t-statistics, since we also observed that in the large sample size limit ordinary t-statistics performed equally well irrespective of M-value or β-value basis (Additional file 4). We also observed that in the large sample size limit, ordinary t-statistics performed similarly if not better than regularized t-statistics (Figure 2 & Additional file 4). Thus, given that β-values are more directly interpretable and that in this work we are mostly interested in the performance for relatively large sample sizes, we henceforth focus mainly on β-values.

### Variance filtering (VF) can significantly reduce the PPV in the setting of small effect sizes

Next, we evaluated the effect of filtering CpGs based on variance prior to performing the supervised analysis. Variance filtering has been a very popular feature selection tool in gene expression studies (see e.g. [27,38]), and it has been shown to improve the detection rate of true positives [21,27]. Hence, we asked if the same result holds in the context of DNA methylation data.

**Figure 3:**
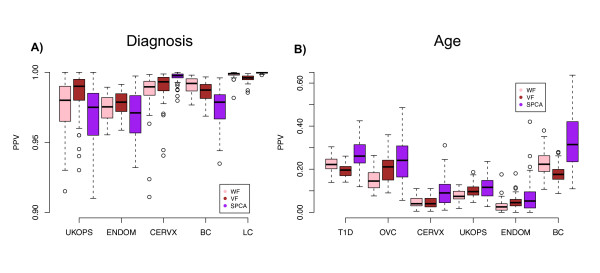
**PPV performance comparisons of all three feature selection methods: without-filtering (WF, pink), variance-filtering (VF, brown), and supervised-PCA (SPCA, purple) on beta-value methylation profiles**. **A) **diagnostic setting in UKOPS, ENDOM, CERVX, BC and LC with the top 200, 1500, 2500, 1500, and 3000 diagnosis-associated-CpGs as the evaluation sets respectively; and **B) **age setting in T1D, OVC, CERVX, UKOPS, ENDOM, and BC with the top 1000, 500, 200, 500, 200, and 1500 age-associated-CpGs as evaluation sets, respectively. A total of 50 training-test set partitions were used.

**Figure 4:**
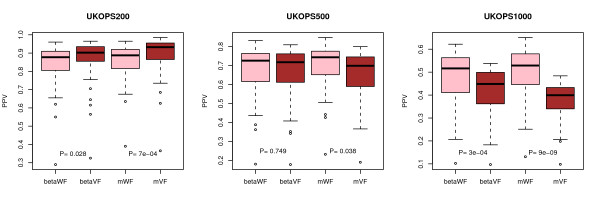
**Comparison of PPV (cancer diagnosis) between the method without-filtering (WF, pink) with variance-filtering (VF, brown) in the UKOPS data set**. The PPV values shown are those obtained from beta-values. PPV values in test sets are shown for evaluation set sizes consisting of the top 200, 500, and 1000 diagnosis-associated-CpGs in the training sets. A total of 50 training-test set partitions were used. All p-values shown are from a Wilcoxon-rank sum test.

We observed that filtering based on variance improved the PPV in 3 of the 5 cancer/normal studies and in 3 of the 6 studies when age was the phenotype of interest (Figures 3A and 3B). In the age-setting, VF did worse than WF in two studies by approximately 5% in PPV values, while in the diagnostic setting the worse performance observed in two studies was far less marginal (change in mean PPV < 1%), indicating that in cases where effect sizes and signal strengths are small (i.e. age), filtering based on variance can be counterproductive. Thus, we posited that in the case of cancer diagnostic markers in whole blood (where signal strengths are relatively large but effect sizes are small), that VF would also lead to a decreased PPV performance if larger evaluation sets are used since the larger evaluation sets would include proportionally more false positives (on account of the small effect sizes). Similarly, we would expect that for larger evaluation sets the WF method would allow proportionally more true positives to be captured. Confirming this, we observed that WF outperformed VF as the number of top ranked CpGs is increased (Figure 4). This switch in performance between WF and VF as a function of evaluation set size is a consequence of the relatively small effect sizes combined with the relatively large signal strengths of diagnostic markers in whole blood.

### SPCA outperforms WF and VF when effect sizes are small

Supervised principal components has been shown to be a simple yet powerful algorithm for performing feature selection and classification in the gene expression context [21]. We therefore decided to compare SPCA to the WF and VF methods in terms of feature selection. Interestingly, in the diagnostic setting SPCA did not always outperform VF or WF, although in all data sets it achieved very high (> 95%) PPV values (Figure 3A). However, in the more challenging scenario of small effect sizes and signal strengths, as illustrated by using age as the phenotype of interest, we can see that SPCA achieved substantially better PPV values (Figure 3B). Thus, principal components can identify true positives more reliably when signal strengths are small. On the other hand, if signal strengths are large, a principal component may not have the plasticity to capture all the important true positives, and thus inevitably will include false positives among the less weighted features within the principal component (PC). We verified that in the diagnostic setting, SPCA PPV values were substantially higher and comparable to those of WF and VF when using only the top 50 CpGs in the selected PC (Additional file 5), demonstrating that at more relaxed feature selection thresholds SPCA includes numerous false positives

### Signal correlation structure is improved using beta values

**Figure 5:**
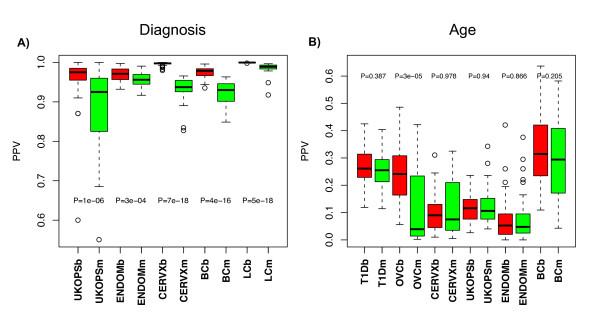
**SPCA PPV performance comparison of beta-values (red) with m-values (green)**. **A) **Diagnostic setting in UKOPS, ENDOM, CERVX, BC and LC with the top 200, 1500, 2500, 1500, and 3000 diagnosis-associated-CpGs as evaluation sets, respectively; and **B) **Age setting in T1D, OVC, CERVX, UKOPS, ENDOM, and BC with the top 1000, 500, 200, 500, 200, and 1500 age-associated-CpGs as evaluation sets, respectively. A total of 50 training-test set partitions were used. All p-values shown are from a Wilcoxon-rank sum test.

Given that overall feature selection was optimal using SPCA, we next asked if performance would be similar had we used M-values. Remarkably, we observed that SPCA PPV values were generally higher when evaluated using beta values (Figure 5). This was specially true in the diagnostic setting and was independent of tissue type, indicating that signal strength has a major impact on the inference of biologically relevant principal components. Interestingly, therefore, beta-values provide a basis in which the correlative structure of CpG methylation profiles associated with cancer status is better preserved. In contrast, in the M-value basis, it appears that the correlation structure between biologically relevant CpGs is compromised leading to worse modelling of the biological variation.

### The elastic net and SVMs outperform SPCA and LASSO

Next, we turned our attention to the classification task. We considered a total of four different powerful classification algorithms, including SPCA, LASSO regression, the Elastic Net (ELNET) and support vector machines (SVM) (Methods). We used the same training-test set partition strategy as with our feature selection analysis. Age was chosen as the phenotype of interest, since it is now well established that age has an impact on DNAm patterns [3-5,42] and because age-associated effects are of a relatively small magnitude, thus also providing a more challenging evaluation scenario for the classification algorithms [3-5]. Moreover, age-associated DNAm markers may represent cancer risk markers [3,6]. We considered the same studies as those used for the feature selection analysis in the age setting.

**Figure 6:**
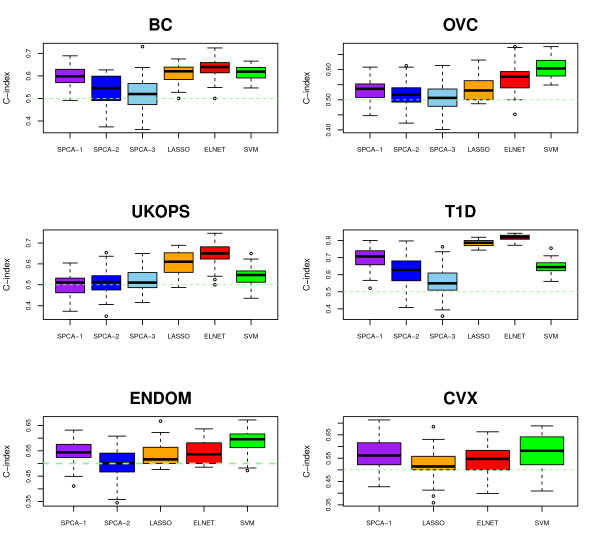
**Classification method comparison of SPCA1, SPCA2, SPCA3, LASSO, ELNET and SVM, using age as the phenotype of interest, for each of the 6 DNAm studies with available age information**. A total of 50 training-test set partitions were used, so each boxplot contains 50 data points. C-index (y-axis) measures the concordance index between the DNAm age predictor score and age itself. Dashed green line denotes the null hypothesis of no association.

Across all studies, we observed that either ELNET or SVM were superior to LASSO and SPCA (Figure 6). The fact that ELNET outperformed LASSO in all data sets extends previous results obtained on gene expression [32] to the DNA methylation context. Moreover, we observed that SPCA always performed optimally in the context of a single component (SPCA-1), i.e. adding more principal components to the classifier did not improve performance (Figure 6). Interestingly, SPCA-1 performed similarly to ELNET and SVM in the two smaller studies (ENDOM + CVX), suggesting that the rigidity imposed by principal components can be of an advantage in this setting.

### Unsupervised modelling of diagnostic and age effects

**Figure 7:**
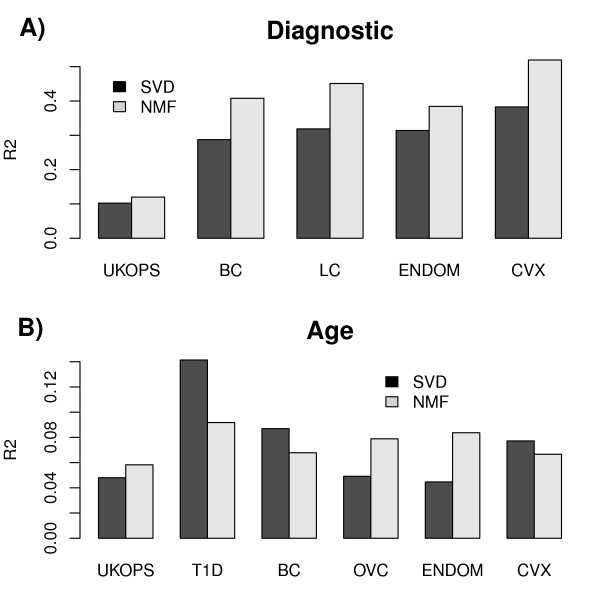
**Unsupervised modelling of A) diagnosis and B) age**. We compare the average R^2 ^values (y-axis) of the best two components correlating with the phenotype of interest across the studies (x-axis) as indicated. NMF was initialised with non-negative double SVD to allow direct comparison to SVD.

Finally, we compared unsupervised algorithms in their ability to model diagnostic and age effects in DNA methylation studies. We considered two of the most popular unsupervised dimensional reduction algorithms from the gene expression field: singular value decomposition (SVD) [43-46], and non-negative matrix factorization (NMF) [47-51]. Given that DNA methylation beta-valued data are defined on the compact support (0,1) and are thus positively valued, it is justified to explore the application of NMF in this context. To objectively compare NMF to SVD, NMF was initialised using non-negative double SVD, thus allowing us to determine if the positivity constraints of the NMF framework add biological value. For both SVD and NMF we computed the R^2 ^correlations between the inferred components (or meta-genes) and the phenotype of interest. As a modelling criterion we compared the average R^2 ^of the two best components, obtained from each algorithm. Focusing first on the diagnostic setting, we can clearly observe that NMF outperformed SVD/PCA in all 5 studies, including the study comparing blood samples from cancers and controls (Figure 7). However, NMF was not conclusively better in the age-setting of much smaller effect sizes (Figure 7).

## Discussion

A previous study compared β to M-values in the context of a titration experiment using fold-change values to rank CpGs [19]. The study concluded that M-values, owing to their reduced heteroscedasticity, provided a better basis in which to detect true positives. This result is not unexpected because fold changes only consider the magnitude of differences in mean values, regardless of the underlying biological and technical variability. Therefore, using β-values and fold changes, true positive CpGs with low or high mean beta methylation values would not be highly ranked and would result in a significant proportion of false negatives. As we have shown here, using many different data sets and using a multiple training test set strategy to estimate the PPV, t-statistics derived from M-values and β-values are comparable and lead to similar PPV values, but only in the scenario of relatively large sample sizes. In contrast, when sample sizes are small and a regularized t-statistic must be used, M-values provided a significantly better basis for inference, sometimes by as much as 10% improvements in the PPV. That regularized t-statistics perform relatively poorly in the β-value basis is not surprising, since the regularisation involves an empirical Bayes approach in which a posterior variance is estimated from estimates specifying the prior, an approach which is known to be more sensitive to the precise distributional properties of the data and is therefore also more sensitive to deviations from normality. Therefore, our comprehensive study using statistics to rank features, indicates that the severe heteroscedasticity of β-values does not compromise the reliability of identifying true positives in the case of relatively large sample sizes, but that M-values offer significantly improved inference in the limit of small sample sizes.

A second important finding is that variance filtering does not always increase the PPV. Indeed, in scenarios where effect sizes are small but where there are numerous associated features, we have seen how variance filtering can compromise the detection of true positives when more relaxed significance thresholds are used. This was illustrated in the context of cancer diagnostic methylation markers in whole blood, where the changes reflect underlying changes in blood cell type composition as demonstrated by us previously [11]. In this setting, effect sizes are small but may involve a potentially large number of genes, indeed potentially all those genes whose expression differentiates blood cell types from each other and whose expression is under epigenetic regulation. Since very often there is a desire to perform Gene Set Enrichment Analysis [54] on a set of top ranked features, thresholds must be chosen to ensure a relatively small false negative rate (FNR). Hence, in the scenario described here, variance filtering may be specially counterproductive since it leads to a larger FNR and hence could compromise the detection of enriched GO-terms or pathways. It follows that any decision to filter features based on variance must take into consideration the clinical and biological context of the study.

Our third key result is the improved performance of SPCA in detecting true positives, specially in the more challenging scenario when effect sizes and signal strengths are small. While SPCA has been shown to outperform many other feature selection methods in the context of gene expression data [21], an analogous result was still lacking in the case of DNA methylation. Our data therefore show that SPCA is a powerful feature selection algorithm, independently of the molecular profiles considered or their underlying statistical distributions. The improved performance over VF also indicates that the correlative DNAm patterns can be exploited to identify true positives more reliably. Interestingly, we observed that SPCA worked better in the context of β-values. This may be surprising as one might be inclined to believe that components of maximal variation are more robustly identified in a basis that promotes variation, i.e. using M-values. However, it is also the case that M-values would aggravate the effect of outliers and therefore distort the true components of maximal biological variation. Therefore, Illumina's recommendation to use β-values over M-values appears to be further justified by the fact that β-values, by virtue of being bounded, may provide a natural regularization and thus suppress the undesirable effects of potential technical outliers.

We also compared various powerful classification algorithms, including SPCA, ELNET, LASSO and SVMs. Our results clearly indicate that ELNET and SVM are superior classification methods in the case where effect sizes are small. Given that ELNET also provides an automatic means of feature selection (i.e. those features with non-zero regression coefficients), it would appear to be a preferable choice over SVM. Nevertheless, SVM outperformed ELNET in three studies, including the two smaller ones (ENDOM + CVX). Interestingly, we also observed that classification using SPCA performed optimally in the context of a single component of variation (SPCA-1). Since multi-component SPCA (SPCA-2, 3) uses a training set to estimate an optimal linear combination of principal components that then makes up the predictor, it would appear that the estimated weights specifying the linear combination is not mirrored in the test set, hence why SPCA-1 is optimal. It will be interesting to investigate if this result generalizes to other phenotypes where effect sizes may be small.

Finally, we also compared two of the most popular unsupervised algorithms (SVD and NMF) in their ability to model differences between cancer and normal tissues and differences associated with age. Similar to the results obtained on gene expression, we observed that NMF also outperforms SVD/PCA in the context of DNA methylation data. Indeed, the meta-genes inferred from the NMF were generally more highly correlated with cancer/normal status across all data sets considered. Hence, despite the increased computational complexity of NMF, this algorithm should be used whenever expected effect sizes are large. On the other hand, if effect sizes are small (age-setting) we did not find that the increased computational complexity of NMF offered any advantage over PCA.

In the context of evaluating feature selection and classification methods, it is important to discuss the impact of potential batch effects. Indeed, as shown in many previous papers [55-59], known and unknown batch effects can affect a substantial proportion of features in an experiment and lead to biased estimates of statistical significance. In this work we used inter-array normalised data where beadchip effects and variations in bisulfite conversion efficiency were adjusted for using a linear model framework, as these factors were observed to account for significant components of variation in PCA analysis (see e.g. [11]). We observed that not correcting for these factors may alter the absolute performance of any given method, yet the relative performance of the different methods was largely robust to whether inter-array normalisation was performed or not (data not shown). Since the inter-array normalisation used only adjusts for known effects (i.e. beadchip and bisulfite conversion efficiency), we have not considered here the potential effects of unknown confounders. To address this would require adapting Surrogate Variable Analysis (SVA) approaches [55,59] in the training/test set evaluation framework considered here. This has only very recently made possible through an extension of SVA, called frozen SVA [60]. Thus, it will be interesting to further compare these feature selection methods in the context of the present DNA methylation data sets, and thus further assess the potential impact of (unknown) batch effects on feature selection and classification.

While the insights obtained in this study have been derived from one particular array (the 27k Infinium beadchip), it is likely that similar insights would apply to the recently released scaled up 450k Infinium beadchip [10,35]. We should point out however, that one of the key differences between the 27k and 450k platforms is that the probes on the 450k beadarray come in two different designs and are thus characterised by widely different statistical distributions [61]. Thus, the insights obtained here are likely to apply only to the data restricted to probes of one particular design. In any case, the much higher density of the 450k array (over 480,000 features compared to ~27,000), means that feature selection in 450k data will be even more critical.

## Conclusions

The optimal choice of methylation measure and feature selection method in the context of 27k DNA methylation data is dependent on the sample size, expected effect sizes and signal strength of the specific study. While SPCA is a powerful feature selection tool in DNA methylation studies where effect sizes and signal strengths are small, the Elastic Net and SVMs generally outperform SPCA in the context of classification. These insights are important for anyone embarking on large scale DNA methylation profiling using 27k or 450k beadarrays.

## Abbreviations

DNAm: DNA methylation; PCA: Principal components analysis; SVD: Singular value decomposition; SPCA: Supervised principal components; ELNET: The elastic net; SVM: Support vector machines; NMF: Non-negative matrix factorisation.

## Competing interests

The authors declare that they have no competing interests.

## Authors' contributions

JZ and AET performed the statistical analyses. MW contributed samples and obtained funding. AET wrote the manuscript with contributions from JZ. All authors read and approved the final manuscript

## Supplementary Material

Additional file 1 **Typical scatterplots of mean methylation (x-axis) vs standard deviation in methylation (y-axis) using beta (left panels) and M values (right panels)**. Green line denotes a mean loess smoother. **A) **The blood samples from the 148 healthy controls in the UKOPS study. **B) **The 187 blood samples from the T1D study. Click here for file

Additional file 2 **False discovery rate (FDR) estimation table at different thresholds of top cancer diagnostic associated CpGs obtained from the supervised analysis (without filtering) in the UKOPS, ENDOM, and CERVX data sets**. Click here for file

Additional file 3 **The evaluation set size (y-axis) is plotted against the mean PPV (x-axis) for the different studies. The mean PPV represents an average over the 50 training-test partitions and over the 3 feature selection methods (WF, VF and SPCA)**. **A) **Diagnostic setting, **B) **Age setting. The selected evaluation set sizes in each study and for each phenotype of interest are marked in red. Note that in the case of age, differences in mean PPV between studies could not be minimized because of the additional constraint of a reasonable minimum set size. Click here for file

Additional file 4 **A-B) Comparison of positive predictive values (PPV) obtained from beta (red) and m-values (green) using the without-filtering method and using 50 training-test set partitions (each boxplot contains 50 data points)**. **A) **Diagnosis setting in UKOPS, ENDOM, CERVX, BC and LC with evaluation set sizes of top 200, 1500, 2500, 1500, and 3000 diagnosis-associated-CpGs, respectively; and **B) **Age setting in T1D, OVC, CERVX, UKOPS, ENDOM, and BC with the top 1000, 500, 200, 500, 200, and 1500 age-associated-CpGs as evaluation sets, respectively. **C-D) **Comparison of absolute t-statistics obtained from beta and m-values. **C) **Diagnostic setting in UKOPS, ENDOM, CERVX, BC and LC; and **D) **age setting in T1D, OVC, CERVX, UKOPS, ENDOM, and BC. All p-values shown are from a Wilcoxon-rank sum test. Click here for file

Additional file 5 **Mean PPV values between SPCA, WF and VF in the diagnostic setting but using only the top ranked 50 CpGs as evaluation sets**. Click here for file
