# Supplementary figures and images for: A comparison of feature selection and classification methods in DNA methylation studies using the Illumina Infinium platform
Source: BMC Bioinformatics. 2012 Apr 24;13:59. doi: 10.1186/1471-2105-13-59 (PMC3364843; doi:10.1186/1471-2105-13-59)

**A)****Beta-values**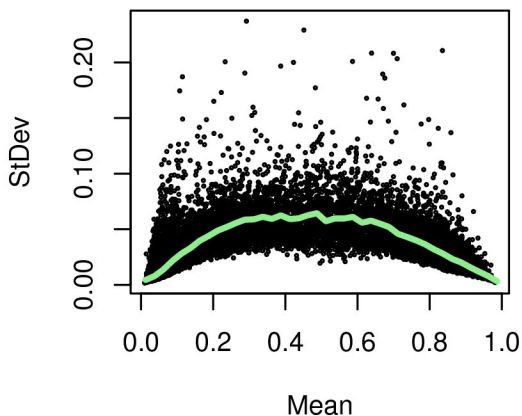**M-values**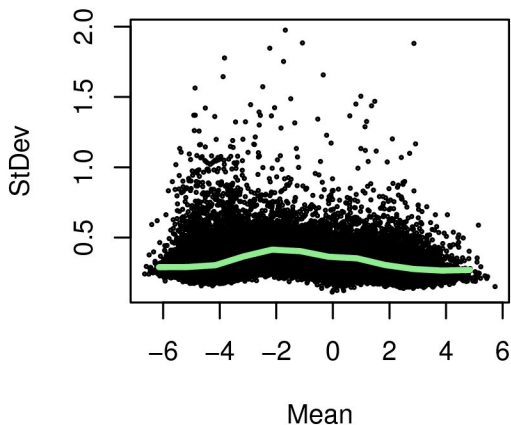**B)****Beta-values**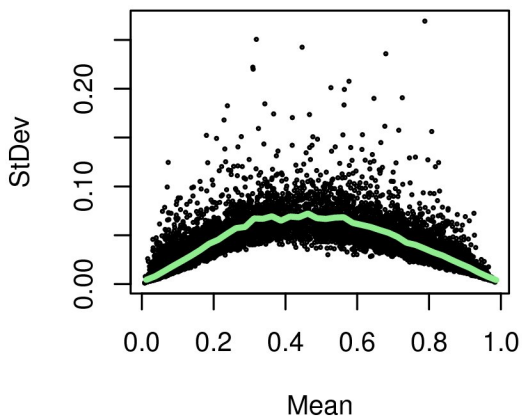**M-values**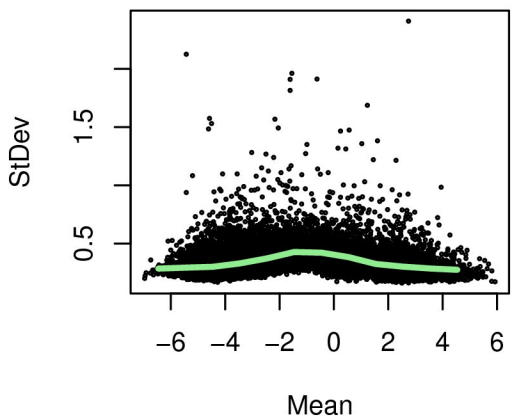

Supplement: Additional file 1 — Typical scatterplots of mean methylation (x-axis) vs standard deviation in methylation (y-axis) using beta (left panels) and M values (right panels). Green line denotes a mean loess smoother. A) The blood samples from the 148 healthy controls in the UKOPS study. B) The 187 blood samples from the T1D study. [file 1471-2105-13-59-S1.PDF]

A)

## Diagnosis

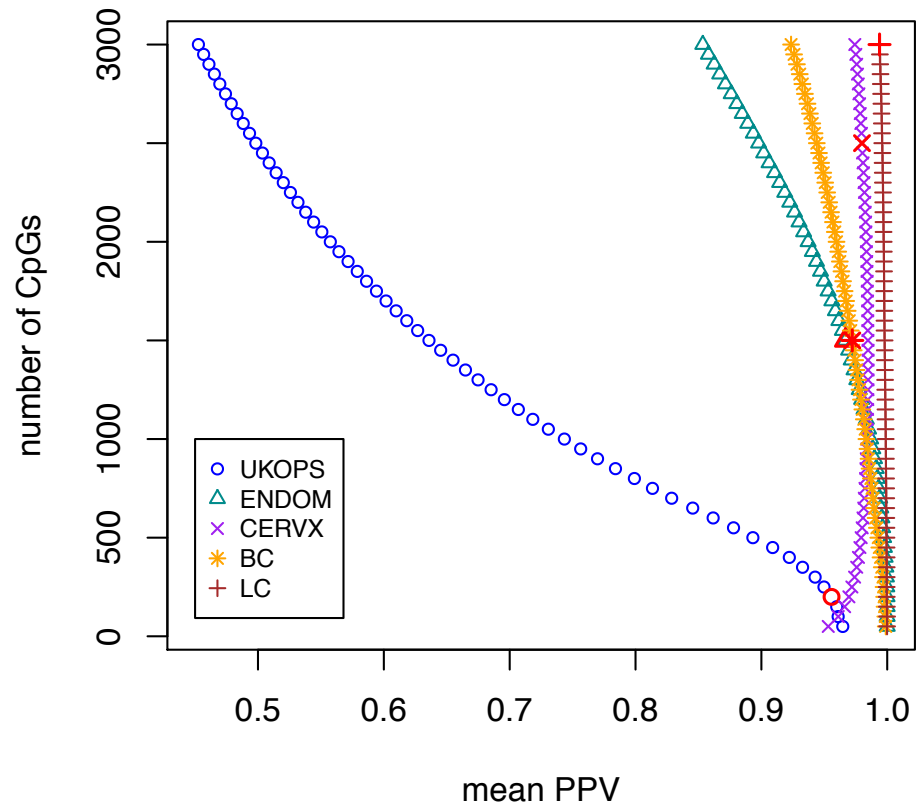

B)

## Age

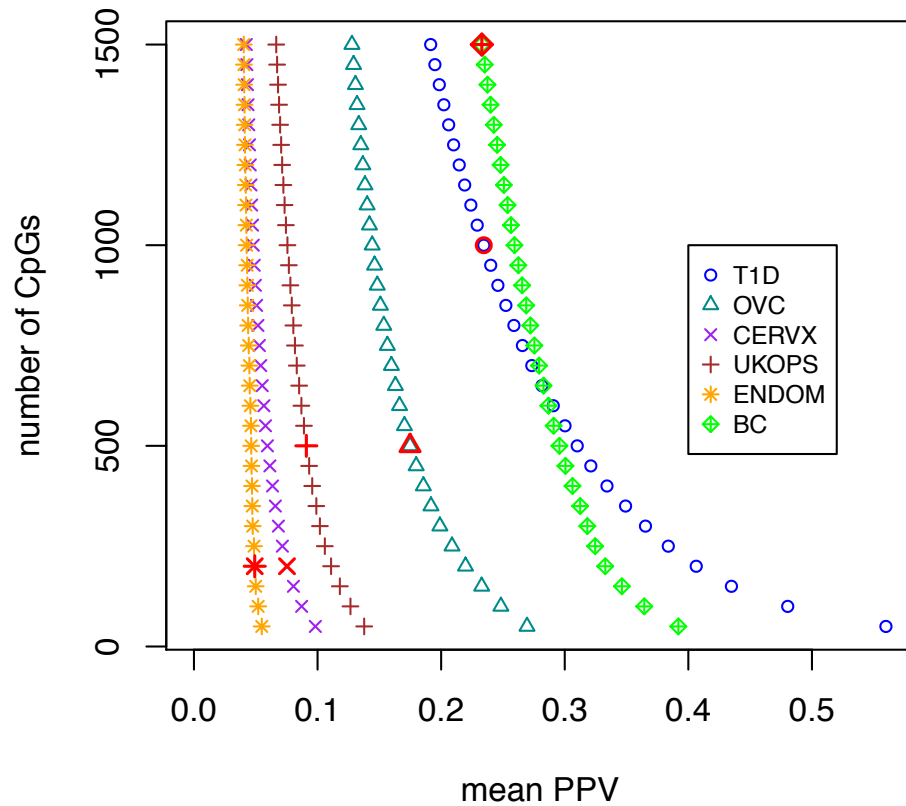

Supplement: Additional file 3 — The evaluation set size (y-axis) is plotted against the mean PPV (x-axis) for the different studies. The mean PPV represents an average over the 50 training-test partitions and over the 3 feature selection methods (WF, VF and SPCA). A) Diagnostic setting, B) Age setting. The selected evaluation set sizes in each study and for each phenotype of interest are marked in red. Note that in the case of age, differences in mean PPV between studies could not be minimized because of the additional constraint of a reasonable minimum set size. [file 1471-2105-13-59-S3.PDF]

# Diagnosis

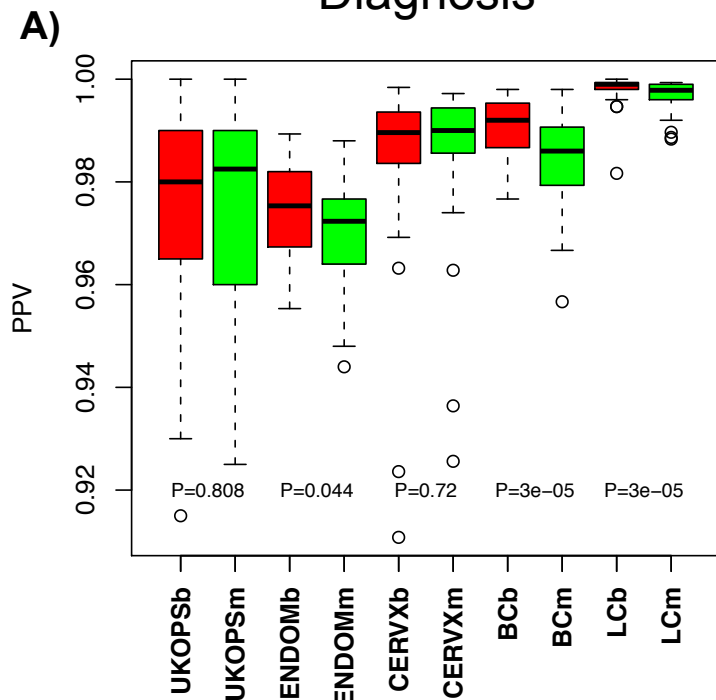

# Age

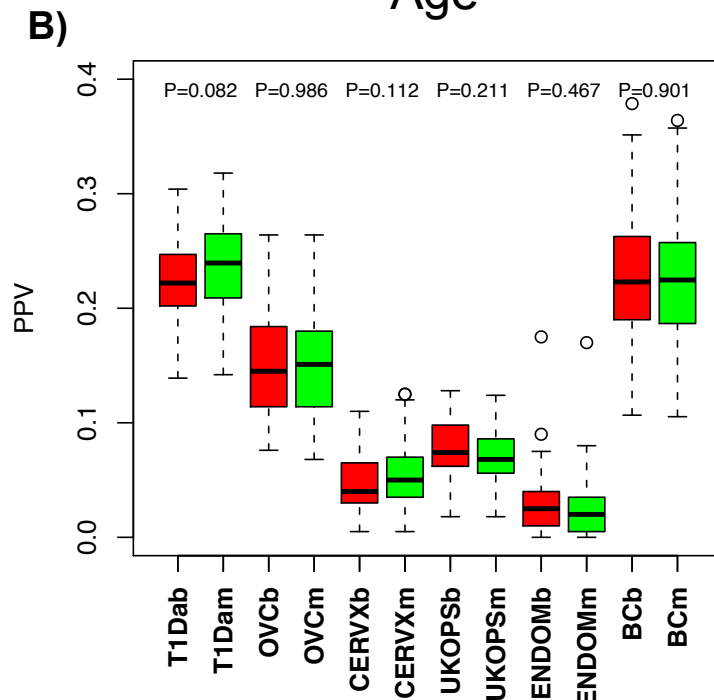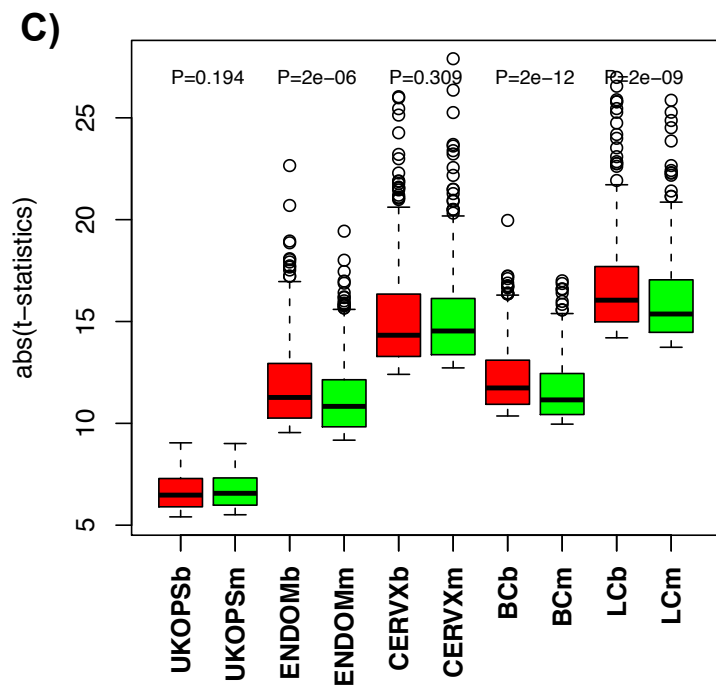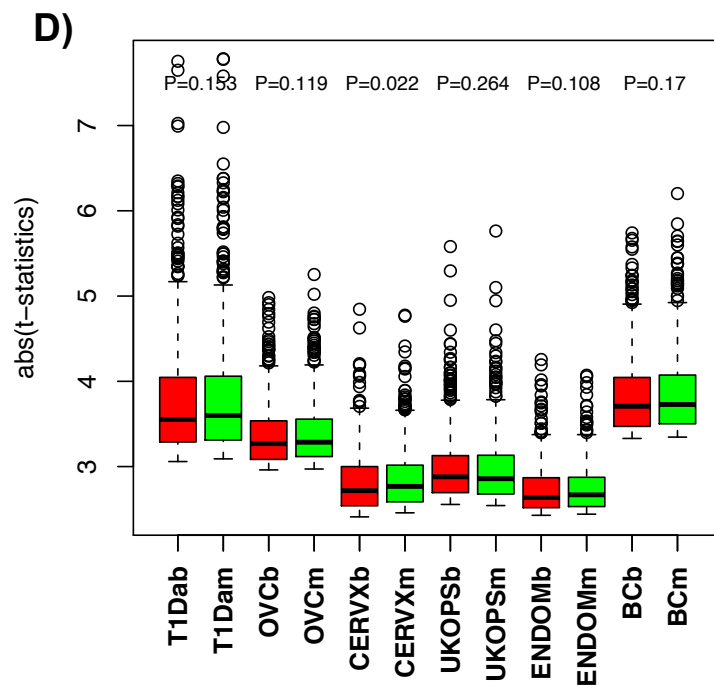

Supplement: Additional file 4 — A-B) Comparison of positive predictive values (PPV) obtained from beta (red) and m-values (green) using the without-filtering method and using 50 training-test set partitions (each boxplot contains 50 data points). A) Diagnosis setting in UKOPS, ENDOM, CERVX, BC and LC with evaluation set sizes of top 200, 1500, 2500, 1500, and 3000 diagnosis-associated-CpGs, respectively; and B) Age setting in T1D, OVC, CERVX, UKOPS, ENDOM, and BC with the top 1000, 500, 200, 500, 200, and 1500 age-associated-CpGs as evaluation sets, respectively. C-D) Comparison of absolute t-statistics obtained from beta and m-values. C) Diagnostic setting in UKOPS, ENDOM, CERVX, BC and LC; and D) age setting in T1D, OVC, CERVX, UKOPS, ENDOM, and BC. All p-values shown are from a Wilcoxon-rank sum test. [file 1471-2105-13-59-S4.PDF]
